# Diagnostic Approach to Pulmonary Hypertension in Premature Neonates

**DOI:** 10.3390/children4090075

**Published:** 2017-08-24

**Authors:** Vasantha H. S. Kumar

**Affiliations:** Division of Neonatology, Department of Pediatrics, The Women & Children’s Hospital of Buffalo, University at Buffalo, 219 Bryant Street, Buffalo, NY 14222-2006, USA; vkumar3@buffalo.edu; Tel.: +1-716-878-7673; Fax: +1-716-878-7945

**Keywords:** pulmonary hypertension, bronchopulmonary dysplasia, premature newborns, nitric oxide, sildenafil

## Abstract

Bronchopulmonary dysplasia (BPD) is a form of chronic lung disease in premature infants following respiratory distress at birth. With increasing survival of extremely low birth weight infants, alveolar simplification is the defining lung characteristic of infants with BPD, and along with pulmonary hypertension, increasingly contributes to both respiratory morbidity and mortality in these infants. Growth restricted infants, infants born to mothers with oligohydramnios or following prolonged preterm rupture of membranes are at particular risk for early onset pulmonary hypertension. Altered vascular and alveolar growth particularly in canalicular and early saccular stages of lung development following mechanical ventilation and oxygen therapy, results in developmental lung arrest leading to BPD with pulmonary hypertension (PH). Early recognition of PH in infants with risk factors is important for optimal management of these infants. Screening tools for early diagnosis of PH are evolving; however, echocardiography is the mainstay for non-invasive diagnosis of PH in infants. Cardiac computed tomography (CT) and magnetic resonance are being used as imaging modalities, however their role in improving outcomes in these patients is uncertain. Follow-up of infants at risk for PH will help not only in early diagnosis, but also in appropriate management of these infants. Aggressive management of lung disease, avoidance of hypoxemic episodes, and optimal nutrition determine the progression of PH, as epigenetic factors may have significant effects, particularly in growth-restricted infants. Infants with diagnosis of PH are managed with pulmonary vasodilators and those resistant to therapy need to be worked up for the presence of cardio-vascular anomalies. The management of infants and toddlers with PH, especially following premature birth is an emerging field. Nonetheless, combination therapies in a multi-disciplinary setting improves outcomes for these infants.

## 1. Introduction

Preterm birth is a major public health challenge leading to significant morbidity and mortality across the globe. Complications of preterm birth are the single largest direct cause of neonatal deaths, responsible for 35% of the world’s 3.1 million deaths a year, and the second most common cause of under-5 deaths after pneumonia [1]. Bronchopulmonary dysplasia (BPD) is the most common chronic respiratory morbidity seen in premature infants, particularly in infants <1500 g [2]. First characterized 50 years ago, BPD was more common in infants >28 weeks gestational age (GA), as infants <28 weeks GA rarely survived lung immaturity and surfactant deficiency. With the advent of antenatal glucocorticoid therapy, surfactant administration and gentle ventilation strategies, BPD is now more frequent in infants <1000 g birth weight or in <28 weeks GA infants. Bronchopulmonary dysplasia has primarily become a function of lung development in extremely low birth weight (ELBW) infants, altered by ventilation, oxygen, chorioamnionitis and genetic factors [2,3,4,5]. Infants with BPD are at increased risk for pulmonary hypertension (PH) with right ventricular failure or chronic lung morbidities contributing to poor outcomes [2,6,7]. Treatment of PH in premature neonates is evolving and there is limited knowledge on how best to treat these infants. Altered lung development can influence airway and vascular growth postnatally affecting not only the pathophysiology of pulmonary hypertension but also the response to treatment with age.

### 1.1. Lung Development

**Figure 1:**
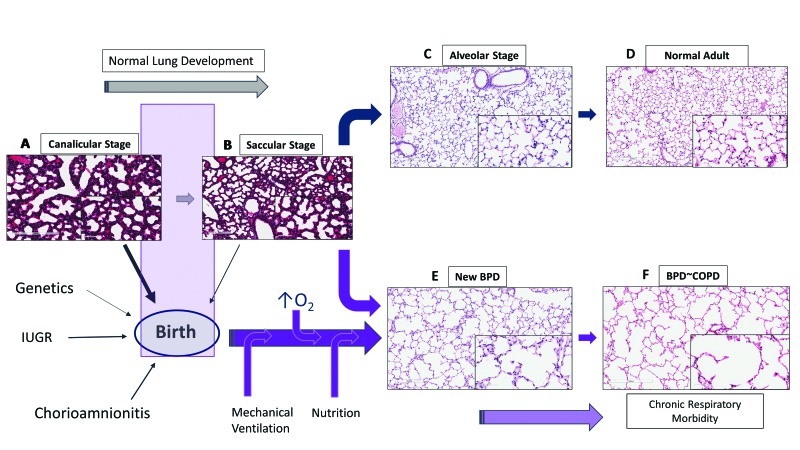
Lung development and bronchopulmonary dysplasia (BPD). Perinatal and postnatal factors play an important role in the development of BPD. The figure illustrates stages of lung development in mice: (**A**) canalicular stage; (**B**) saccular stage; (**C**) alveolar stage; (**D**) lung histology in adult mice; (**E**) new BPD with simplified alveoli and fewer secondary septae in mice exposed to 85% O_2_ from day 3 (P3) to day 14 (P14); (**F**) lung similar to chronic obstructive pulmonary disease (COPD) in adult mice at 9 months following neonatal oxygen exposure. IUGR: intrauterine growth retardation; O_2_: oxygen. (*Slide magnification*—100×; *Inset slides*—400×; See text for details).

Lung maturation occurs through five stages: embryonic (4–7 weeks); pseudoglandular (7–17 weeks); canalicular (17–26 weeks); saccular (27–36 weeks) and alveolar stages (36 weeks–2 years). During late canalicular stage (22–26 weeks), terminal bronchioles become larger and lung tissue becomes vascular with formation of alveolar ducts (Figure 1A). Respiration is possible with some survival in these tiny immature infants as epithelial differentiation is beginning to occur. These are the infants who at most of risk of BPD with chronic respiratory morbidity and mortality. During saccular stage of lung development (27–36 weeks), many more terminal sacs and alveolar ducts form with thinning of the epithelial surface, bulging of the capillary tubes into the alveolar surface and surfactant production beginning to increase with gestational age. Beyond the early stages of saccular period, most of these infants have sufficient surfactant production and adequate air-capillary interphase for gas exchange to survive. However, alveolar formation only begins around 36 weeks and continues after birth up to 4 years of postnatal life [8].

Preterm lungs in late canalicular or early saccular stage of lung development are particularly sensitive to prenatal or postnatal alterations leading to impairment in alveolar and vascular development resulting in BPD (Figure 1). Chorioamnionitis, preeclampsia and intrauterine growth retardation can affect the development of the fetal lung [4,9]. Soon after birth, oxygen administration, mechanical ventilation and cytokine responses from accompanying inflammation or infection and nutritional status of the infant disrupt normal lung development [5], resulting in histologic pattern of fewer and larger alveoli or alveolar simplification termed as new BPD [3].

### 1.2. Lung Development and Angiogenesis

Alveolar capillaries are abundant in mature lungs and are in close proximity to the epithelium, creating an air-blood barrier for optimal gas exchange (Figure 1C). Vasculogenesis, a process of new vessel formation from primitive mesenchymal and endothelial network and angiogenesis, formation of vessels from pre-existing vessels, both are dependent on angiogenic factors such as vascular endothelial growth factor (VEGF) and angiopoietin-1. The interactions between airways and blood vessels is critical for normal lung development, facilitated by a coordinated and timely release of VEGF, promoting alveolar development [10]. Disruption of pulmonary vasculature from altered angiogenesis may be an important component of BPD [11]. VEGF mRNA and protein expression peaks in the canalicular stage of lung development when most of the vessel growth occurs, with localization in the distal airways and airway epithelial cells [12]. The expression decreases after birth in mice until day 10 postnatal (P10), when it plateaus to adult levels [13]. VEGF receptors 1 and 2 (VEGFR1/2) are localized on pulmonary endothelial cells in close proximity to the developing epithelium [14]. In the developing lung, epithelial cells by expressing VEGF regulate the temporal and spatial distribution of capillary network to facilitate optimal gas exchange.

### 1.3. Vascular Dysplasia in BPD

BPD not only results in alveolar simplification with larger alveoli and fewer septae but also results in smaller pulmonary arteries with decreased capillary density (Figure 1E). Although the number of endothelial cells has not been quantified in human BPD, decreased endothelial cells and microvascular dysmorphogenesis are characteristics in an experimental model of BPD in premature baboons [15]. Human infants dying with BPD have abnormal alveolar micro vessels and disordered expression of angiogenic growth factors and their receptors [11]. Hyperoxia by inhibiting VEGF expression during critical period of lung development may contribute to the later development of BPD [16]. Endothelial nitric oxide synthase (NOS), VEGFR1 and VEGFR2 expression are decreased in pulmonary arterial endothelial cells (PAEC) suggesting endothelial dysfunction [17]. Decreased alveolar and vascular growth seen in these infants [17] adversely affect respiratory outcomes such as BPD and PH.

## 2. Bronchopulmonary Dysplasia and Pulmonary Hypertension

**Figure 2:**
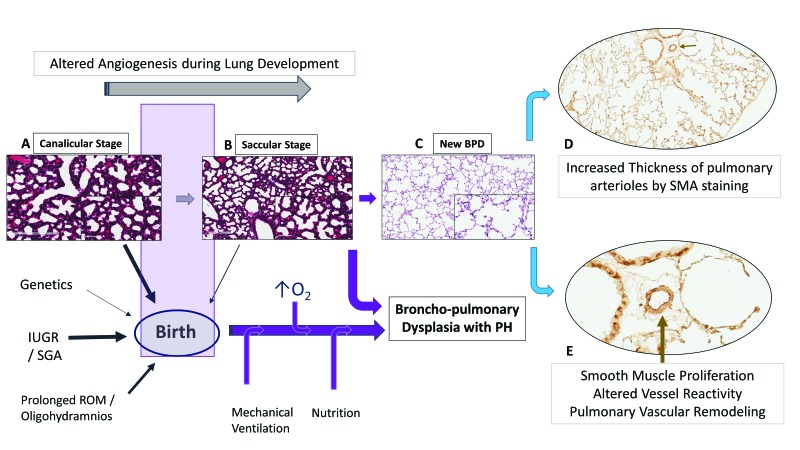
Bronchopulmonary dysplasia and pulmonary hypertension (PH). (**A**–**C**) Altered angiogenesis during lung development in the presence of perinatal and postnatal factors leads to pulmonary hypertension in BPD; (**D**) smooth muscle actin (SMA) staining demonstrating increasing thickness of the pulmonary arterioles following neonatal O_2_ exposure (arrows—Figure 2D); (**E**) high power magnification (400×) illustrating smooth muscle thickness of the pulmonary vessel wall. SGA: small for gestational age. ROM: rupture of membranes.

Bronchopulmonary dysplasia, one of the major complication of prematurity, is the most common form of chronic lung disease seen in a third of ELBW infants [18]; and pulmonary hypertension is noted in a fifth of ELBW infants, primarily in infants with moderate-to-severe BPD [6,7,19] (Figure 2). In the largest meta-analysis to date, the odds of PH was higher in all forms of BPD versus no BPD; the pooled incidence of PH was 4% in mild BPD increasing to 33% in severe BPD [19]. Infants in the meta-analysis were <32 weeks gestation and beyond 4 weeks of postnatal age with confirmed PH, indicating that structural alterations in lung histopathology during development has significant impact on development of PH [19]. As dysregulated vascularization is one of the main features of BPD, it is not surprising that BPD and PH often co-exist. However, the risks of pulmonary hypertension are not always related to severity of BPD; as many infants with moderate-to-severe BPD do not develop PH, indicating that they can occur independent of each other. New BPD may represent a developmental lung disorder, affecting both pulmonary microvascular growth and alveolarization. Alterations in either of the components not only affect the ability of the lung to function as an effective ventilation and oxygenation unit but also determines the degree of vascular resistance in the pulmonary vascular bed. Pulmonary hypertension and BPD parallels lung development and hence are intricately linked to prematurity (Figure 2). An inverse relationship exists between birth weight and cardiopulmonary disease in adults including pulmonary hypertension [20]. Small for gestational age (SGA), oligohydramnios, sepsis, perinatal stress, and prolonged mechanical ventilation are additional risk factors for development for PH in infants [21,22,23,24] (Figure 2). Elevated pulmonary vascular resistance (PVR) in preterm infants with hypoplastic lungs [25] may be related to maldevelopment from a decrease in cross-sectional area and an abnormal muscularization of the pulmonary vasculature [26], exacerbated by the physiologic immaturity of the nitric oxide (NO) signaling pathways [27].

### 2.1. Hyperoxia, Oxygen Saturations, BPD and PH

Oxygen administration in BPD may be the result of initial disease severity, making it difficult to isolate the independent causative effect of inspired oxygen concentration on BPD. However, animal studies support oxidant stress from supplemental oxygen use as one of the factors in the development of BPD [10]. Multiple studies have demonstrated that exposure to hyperoxia during critical periods of lung development, impairs alveolar development with inhibition of secondary crest formation [28,29,30]. In the Supplemental Therapeutic Oxygen for Prethreshold Retinopathy of Prematurity (STOP-ROP) trial, infants in the supplemental group who received oxygen, required higher supplemental oxygen therapy and diuretics at 50 weeks post-menstrual age (PMA), suggesting supplemental oxygen may contribute to BPD [31]. Even though oxygen is a potent pulmonary vasodilator, the short-term benefits of hyperoxia has to be balanced against hyperoxia induced lung injury [32,33]. Pulmonary hypertension, an abnormal physiologic response to a structural alteration in the lung, is often the link between lung dysfunction and the structural and functional adaptation of the right heart. Unique alterations in right ventricular mitochondrial regulation, in a rodent model of postnatal hyperoxia at one year of age [34] suggest long lasting effects on the heart. Mitochondrial DNA damage and dysregulated biogenesis from postnatal hyperoxia could increase the risk for right ventricular (RV) dysfunction in premature infants with BPD [34]. Despite the recently conducted randomized trials comparing different oxygen saturation (SpO_2_) targets, optimal oxygen saturation range in premature infants has remained elusive. Among the three oxygen saturation trials, infants randomized to the higher oxygen saturation group (91% to 95%) had a significantly higher incidence of severe retinopathy of prematurity (ROP), and infants randomized to the lower SpO_2_ group (85% to 89%) had higher mortality at discharge [35,36,37]. Canadian oxygenation trial did not demonstrate any difference in mortality between the low and the high SpO_2_ groups [38]. The ideal physiologic target range of oxygen saturation in ELBW infants is more to be likely patient specific and dynamic and depends on various factors, including gestational age, chronological age, underlying pathology, and transfusion status [39]. Even though the ideal SpO_2_ for ELBW infants remains unknown, it may be safer to maintain an oxygen saturation target of 91% to 95% in infants with BPD or BPD-PH.

### 2.2. Small for Gestational Age Infant, BPD and PH

Increased risk of BPD and increased mortality is seen in premature infants born small for gestational age [40]. Growth retarded premature infants with PH are at significant risk of death [22]. Longitudinal follow-up has shown a trend toward higher morbidity and death among PH infants with birth weight <25th percentile [7] Fetal growth and intrauterine growth retardation (IUGR) are not only important predictors of PH in premature infants with moderate-to-severe BPD, but also can result in varying degree of pulmonary arterial hypertension (PAH) or pulmonary vascular remodeling in later life [41,42]. Disordered vascular growth leading to PH, particularly in growth-restricted infants may have fetal origins. In growth-restricted infants, hypoxic events either in utero or postnatal, may alter pulmonary vasoreactivity and predispose to vascular remodeling in later life (Figure 2). Maternal nutrient restriction increases histone acetylation and hypoxia inducible factor-1α (HIF-1α) binding levels in the endothelin-1 (ET-1) gene promoter of pulmonary vascular endothelial cells (PVEC) in growth-restricted newborn rats for up to 6 weeks after birth [42]. This may predispose growth-restricted newborns to heightened sensitivity to hypoxemic episodes later in life, resulting in pulmonary vascular remodeling. Post-hoc study of the Surfactant, Positive Pressure and Oxygenation Randomized (SUPPORT) trial [36] found evidence of an interaction between SGA and lower oxygen saturation targets (SpO_2_: 85–89%) with increased mortality in these infants [43]. Fluctuating oxygen concentrations alter HIF and angiogenic gene expression [44] and may be an important factor in the development of PH. Neonatal oxygenation prospective meta-analysis may offer additional insights into the effects of growth on postnatal oxygen saturation targets. The findings that epigenetics may be closely associated with the development of hypoxic PAH following IUGR provide new insights in the prevention and treatment of IUGR-related PAH [42].

### 2.3. Vessel Reactivity, BPD and PH

In a murine model of hyperoxia induced BPD-PH, acetylcholine an endothelium-dependent relaxing agent reduced and contractile agents exacerbated pulmonary arterial reactivity [45]. Altered relaxant response of the pulmonary arteries is due to reduced endothelial nitric oxide synthase (eNOS) phosphorylation and enhanced contractility from an increase in cytosolic calcium in pulmonary arterial smooth muscle cells (PASMCs) [45]. Interestingly, Ca^2+^ signaling in PASMC plays a central role both in vasoconstriction, through its pivotal effect on PASMC contraction, and in vascular remodeling, through its stimulatory effect on PASMC proliferation and migration [46]. In mice models, hyperoxia induced lung injury results not only in airway smooth muscle hypertrophy [28], but also in increased thickness of pulmonary arterioles (pulmonary vascular remodeling) three to nine months following neonatal oxygen exposure (Figure 2) [47]. Beyond the neonatal increased pulmonary vascular tone and heightened vasoreactivity may contribute to sustained PH and potentially increase risk for late PH associated with BPD.

Histological assessment of the pulmonary vasculature may provide clues regarding the severity of pulmonary hypertension in infants. Only limited data is available on the histopathological features in premature infants. Significant differences in arterial wall thickness among control, mild and severe chronic lung disease (CLD) infants at autopsy indicate that pulmonary arterial remodeling can progress rapidly with clinical deterioration of the infant [48]. An increase in circumferential actin in the pulmonary vessels, in infants with severe CLD compared to mild disease, suggest that these infants need to be followed closely for PH by echocardiography [48]. Prolonged rupture of membranes and pulmonary hypertension of the newborn (PPHN) worsened the histological changes in these infants [48]. We have shown that preterm infants who died of severe respiratory failure also exhibit muscularized arteries on smooth muscle actin staining [21]; and that the smooth muscle area ratio is directly related to severity of respiratory disease in these infants [21]. As the pulmonary vasculature of the premature neonate has the capacity to remodel over time, a potential exists for the development of PH later on in life, especially so in infants with mild to moderate BPD. Premature and growth restricted infants without BPD are at particular risk for development of PH later on in life. Disorders of fetal growth may heighten the sensitivity of the arterioles to pulmonary vascular remodeling from hypoxemic events in later life, leading to pulmonary hypertension. There is paucity of long-term studies on development of PH in children and adults in former premature infants with no BPD.

Our understanding of the pathophysiology of PH and its association with BPD in the context of lung development is evolving. In a prospective study, right ventricular index of myocardial performance measured serially by echocardiography, a marker of PVR steadily declined in non-BPD infants but not in BPD infants at day 7, 14 and 28 [49]. Of note, these infants were of higher birth weight with a mean GA of 28 weeks. Hyaline membrane disease in these infants is also associated with delayed postnatal circulation adaptation characterized by pulmonary hypertension and prolonged ductal patency [50]. Ligation of the ductus arteriosus is one of the main risk factors associated with PH in ELBW infants [51]. Early pulmonary vascular disease is associated with the development of BPD and echocardiography at seven days of age may be a useful tool to identify infants at high risk for BPD and PH [52]. In a prospective, single-center cohort study, early PH as determined by echocardiography in the second week after birth, was associated with moderate/severe BPD or death at 36 weeks’ PMA [53]. Early diagnosis of PH with screening echocardiography or by serum biomarkers such as B-type natriuretic peptide may help in appropriate management and may decrease mortality in these infants.

### 2.4. B-Type Natriuretic Peptide (BNP)

Plasma BNP is a hormone secreted by the cardiac ventricles under conditions of volume expansion or pressure overload to regulate circulating blood volume [54]. Pro-BNP is cleaved to the biologically active hormone BNP and the inactive N-terminal (NTproBNP), which are both detected and measured in circulation. Circulating levels of BNP correlate with mean pulmonary arterial pressures (mPAP) and are a reliable marker of right heart strain, a significant predictor of morbidity and mortality in adult PH [55]. Elevated BNP is not specific for right or left sided heart disease, but in the absence of left sided heart disease, may prove to be useful adjunct to echocardiography in evaluating right heart stress from PH. Despite the lack of appropriate reference values in children and neonates, preliminary data suggest that BNP levels are useful in diagnosis and management of heart failure and other cardiovascular lesions including pulmonary hypertension in children. The BNP levels are highest in the first two days of life in healthy newborns and gradually decline over time [56]. Gestational age, large shifts in extracellular volume and patent ductus arteriosus impact BNP levels in the first week of life in neonates. Due to the great variability in circulating levels, the diagnostic accuracy of BNP assay might be significantly lower in the first days of life [56]. Higher BMP cutoff may increase specificity for cardiac disease; however, it decreases the clinical usefulness by decreasing sensitivity of this screening tool used in the integrated diagnostic approach in the management of cardiac disease and/or PH.

Plasma BNP is higher overall in neonates with or without significant cardiac disease, than in the older children [57]. The median BNP for neonates (<7 days old) with cardiac disease was 526 pg/mL versus 96 pg/mL for those without disease. The median BNP in older children with cardiac disease was 122 pg/mL versus 22 pg/mL for those without disease. A BNP level of 170 pg/mL has a sensitivity of 94% and a specificity of 73% for significant cardiovascular disease in neonates <7 days old. In neonates >7 days old, a BNP of ≥41 pg/mL predicts a 77% chance of significant cardiovascular disease [57]. The changes in BNP measurements over time significantly correlates with the change in the hemodynamic and echocardiographic parameters in patients with PH. Patients with a BNP value >180 pg/mL had a decreased survival rate [58]. BNP estimation may be useful as a prognostic marker of all-cause mortality in ELBW infants with BPD associated PH [59]. BNP levels may be included in the risk stratification of preterm infants with PH, and higher BNP levels may warrant increased surveillance and appropriate management. However, studies need to address whether incorporating BNP levels in management strategies lead to decreased morbidity and mortality in infants with PH.

### 2.5. PH and Cardiovascular Anomalies

Pulmonary hypertension worsens prognosis for bronchopulmonary dysplasia and the response to therapy with pulmonary vasodilators is neither consistent nor uniform. This may reflect not only the multifactorial nature of BPD but also complex factors at play in infants born prematurely. Cardiovascular anomalies (CVA) noted in infants with BPD and PH further complicates therapy, as not all infants may respond to pulmonary vasodilator therapy. Common CVA noted in these infants include atrial septal defect (ASD), patent ductus arteriosus (PDA), systemic pulmonary collaterals (SPC), and pulmonary vein stenosis (PVS) [60]. Preterm birth is strongly associated with PVS, and 42% of the infants developed BPD prior to diagnosis of pulmonary vein stenosis [61]. The lack of concordance in twins suggests epigenetic or environmental factors may play a role in the development of pulmonary vein stenosis [62]. Factors associated with shorter survival or re-stenosis in infants are stenosis of  ≥3 pulmonary veins, bilateral pulmonary vein stenosis, small for gestational age and <6 months of age at diagnosis [62]. It is interesting to note that many of these patients also have intra-cardiac shunt lesions, which may act in concert with preterm endothelium to produce pulmonary vein stenosis. There appears to be a higher incidence of necrotizing enterocolitis in premature infants with the diagnosis of acquired pulmonary vein stenosis [63]. Systemic to pulmonary collaterals are bronchopulmonary communications that may proliferate in premature infants in response to positive pressure ventilation [64]. Prompt diagnosis for accurate diagnosis of CVA, early shunt closure and aggressive specific drug therapy can improve outcomes in these patients [60]. Presence of moderate-to-severe PH on two successive echocardiograms, worsening of PH in infants with moderate PH and intercurrent infections should necessitate future work-up to rule out CVA. Presence of pulmonary edema may reflect left ventricular (LV) dysfunction or the presence of systemic to pulmonary collaterals; any of these should necessitate cardiac catheterization. The possibility of pulmonary vein stenosis needs ruling out in infants with minimal or lack of response to two pulmonary vasodilators (generally inhaled nitric oxide (iNO) and/or sildenafil). Broadly, the diagnostic work-up includes a multi-slice CT scan initially followed by cardiac catheterization (CC) if needed. This may entail transfer of the patient to a facility that has expertise in performing cardiac catheterization. Cardiac catheterization and CT scan of the chest usually provide information about CV anomalies for both diagnosis and specific therapies. Pulmonary vein stenosis depending on the number of stenotic veins may need stenting and/or transplantation. Percutaneous or surgical closure of shunts such as ASD, PDA or SPC may alleviate PH. Pulmonary vasodilator therapy is helpful in infants with persistent PH who had corrective surgery.

## 3. Diagnostic Approach to BPD with PH

### 3.1. General Work-Up

**Figure 3:**
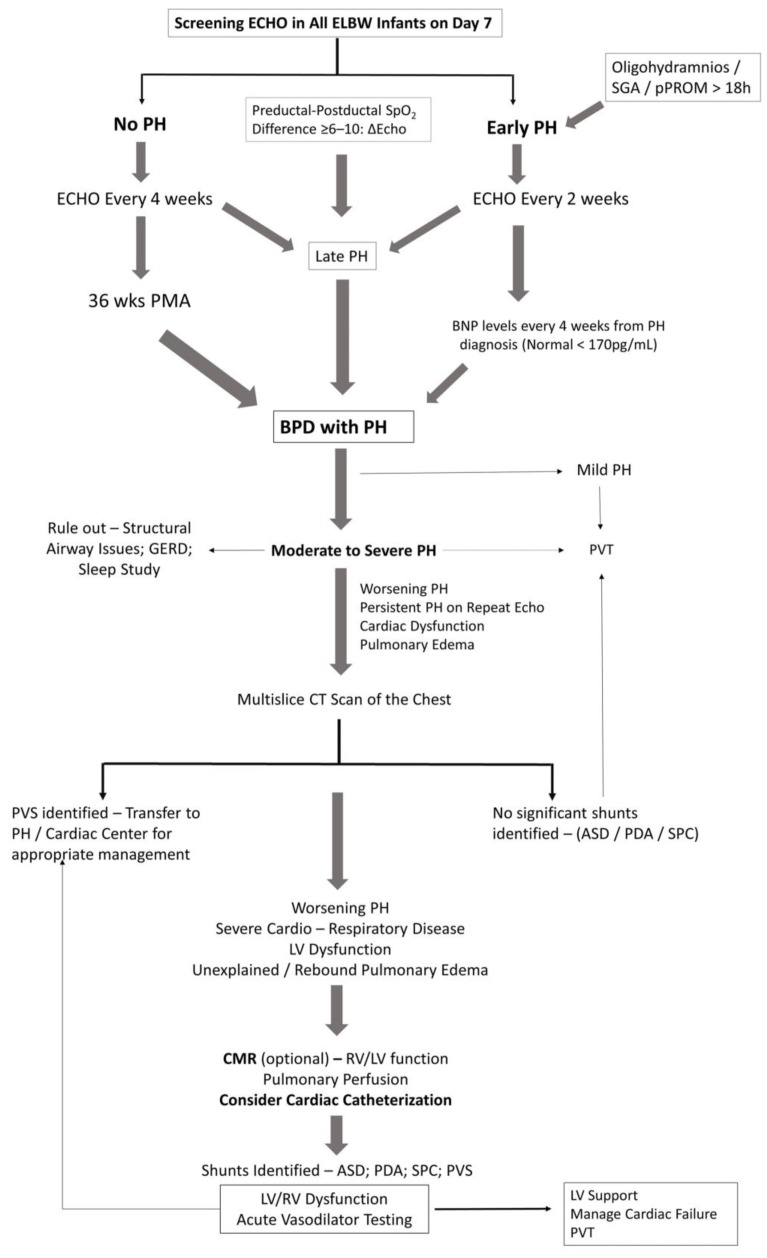
Diagnostic approach to PH in premature neonates. pPROM: preterm prolonged rupture of membranes; Echo: echocardiography; BNP: B-type natriuretic peptide; PMA: post menstrual age; GERD: gastro-esophageal reflux disease; ASD: atrial septal defect; PDA: patent ductus arteriosus; SPC: systemic to pulmonary collaterals; PVS: pulmonary vein stenosis; RV: right ventricle; LV: left ventricle; CMR: cardiac magnetic resonance imaging; PVT: pulmonary vasodilator therapy.

Figure 3 illustrates a broad diagnostic approach for early detection and screening of pulmonary hypertension in extremely premature infants. As our understanding of PH is expanding, so will the diagnostic approach. Physiologic alterations, such as chronic or intermittent hypoxemia or chronic hypercapnia play a significant role in the development of PH in infants with BPD [65]. Continuous monitoring of oxygen saturations and PaCO_2_ assessment in blood gases would help in assessing the risk for PH in infants with BPD. Hypercarbia could represent worsening lung disease from BPD and hence the higher risk for PH. Structural changes in the lung such as tracheomalacia or reactive airway disease resulting from chronic inflammatory changes are not uncommon in infants with BPD, contributing to hypoxemic or hypercarbic episodes. Aspiration of feeds may worsen inflammatory lung disease, worsening PH in these infants [65]. A preductal to post ductal oxygenation difference, in either arterial PO_2_ (≥20 mmHg) or SpO_2_ (≥10%) is significant for pulmonary hypertension in established cases. However, infants with early PH or shunting at the atrial level may not demonstrate pre-post ductal oxygenation gradient. Echocardiography is the main screening tool for the diagnosis of PH in premature infants.

### 3.2. Echocardiography

Transthoracic echocardiography is the most commonly used modality for the diagnosis of PH, as it is both noninvasive and readily available assessment of heart function. Systolic pulmonary arterial pressure (sPAP) is generally equal to right ventricular systolic pressure (RVSP) in the absence of pulmonic stenosis or right ventricular outlet obstruction. An estimate of RVSP is obtained by calculating the gradient between right ventricle and right atrium during systole using Bernoulli’s equation, 4*v*^2^, where *v* is the velocity of the tricuspid jet (m/s) measured by echocardiography. RVSP is derived by adding right atrial pressures (RAP) to the gradient (RVSP = 4*v*^2^ + RAP) [66]. The measure of tricuspid regurgitant jet velocity (TRJV) of <50%, 50–75% or >75% of systemic arterial pressures may provide a clue to the severity of pulmonary hypertension. Semi-quantitative measurements such as right to left shunting of blood through the PDA or foramen ovale are additional findings. Qualitative measurements such as intraventricular septal flattening at end systole, right ventricular hypertrophy and right ventricular dilatation are additional clues, especially in the absence of measurable tricuspid regurgitant (TR) jet. Documenting all the quantitative and qualitative variables will go a long way in establishing an accurate diagnosis of PH, especially while interpreting sequential echoes [67]. Functional assessment of RV is important, as RV function is more predictive of survival in PH than PVR [68]. Use of objective measures of RV function such as tricuspid annular planar systolic excursion (TAPSE) has good correlation with RV function and correlates with survival in children [68].

The reliability of Doppler echocardiographic techniques in detecting and quantifying PAH is pivotal in evaluating its usefulness as a diagnostic procedure and especially as a screening tool. The TR jets are analyzable in only 39% [69] to 86% of patients [70] with PH. The correlation coefficients between RVSP estimated from TR and hemodynamic right-heart catheterization (RHC) values varies widely from 0.39 to 0.90 in adult studies [66]. In children, using TR gradient of >40 mmHg to signify PH, echocardiography demonstrates an 88% sensitivity and 33% specificity to catheterization evidence of PH [71]. Echocardiography was able to detect a measurable TRJV in 61% of patients and estimates of sPAP had both poor correlation and poor accuracy for determining PH severity compared with values measured on subsequent catheterization [71]. Factors such as marked pulmonary hyperinflation, expansion of the thoracic cage and alteration of the position of the heart especially in mechanically ventilated infants may adversely affect the ability to detect and measure TR jet velocity [72]. Poor acoustic windows, operator dependency and the complex geometry of the RV, makes assessment of RV flow velocity with two-dimensional echocardiography difficult. Because the TR jet is not always present or measurable, qualitative echocardiographic measures such as right atrial enlargement, right ventricular hypertrophy or dilation, PA dilation, and septal flattening may improve accuracy of echocardiography as a screening tool in the diagnosis of PH [71]. Despite limitations and inter-observer variability, findings on echocardiography strengthen the diagnosis of PAH and is the most appropriate screening tool for noninvasive diagnosis of PH; however, CC will eventually be required for accurate assessment of PVR to confirm PH.

### 3.3. Magnetic Resonance, CT and PH

A more comprehensive assessment of the RV and the pulmonary vasculature is possible by cardiac magnetic resonance (CMR) and CT [73]. CMR offers the ability to assess the functional and structural features such as ventricular function, blood flow, pulmonary perfusion and myocardial tissue characteristics of the heart. The main role of CT is to detect alternate causes of PH in infants and children such as lung parenchymal disorders, thromboembolic disorders and vascular anomalies such as pulmonary vein stenosis [73].

CMR is a powerful predictor of outcome in adult PH [74]. Traditional CMR requires breath holding and hence are technically difficult to perform in infants and young children. Free breathing cine CMR can be achieved by signal averaging or real-time techniques. The advantage of real time CMR is that it can be safely done without general anesthesia in infants and children who are not sedated and breathing spontaneously. Cine CMR is the gold standard for the assessment of biventricular volumes, muscle mass and global pump function. It is ideal suited to detect changes in RV dimensions and function. In a recent study, RV ejection fraction, and LV stroke volume index were strongly predictive of survival in children with PH, with a 2.6- and 2.5-fold increase in mortality, for every one standard deviation decrease in these indices on CMR [75]. Usefulness of cine CMR was also established in another study, wherein abnormal RV parameters were also noted in children with PH in contrast with healthy children [76]. Assessment of cardiac blood flow by phase contrast magnetic resonance and anatomic assessment by contrast-enhanced CMR angiography are other useful modalities for the assessment of structure and function in children with PH [73]. CMR-augmented cardiac catheterization may offer precise calculation of PVR due to reliable quantification of pulmonary blood flow compared to thermodilution techniques or oximetry [77]. As RV function is the best predictor of outcome, future studies need to highlight the validity of CMR in PAH. This may help to clarify whether routine use of CMR, would improve outcomes especially in infants and children with PH. CMR is limited to specialized centers managing pediatric PH and infants must be transferred to these centers for imaging and management, which is a significant disadvantage for patients and families.

### 3.4. Cardiac Catheterization

Cardiac catheterization is the gold standard for the diagnosis of pulmonary hypertension in infants and children. Catheterization not only defines the hemodynamic profile to confirm diagnosis of pulmonary hypertension but also facilitates vasoreactivity testing and to formulate specific treatment plans for the infant. Indications for catheterization are not clear, especially in infants with BPD associated PH. Nonetheless, findings on either CT scan suggestive of cardiovascular anomalies, significant PH requiring prolonged or combination pulmonary vasodilator therapy and PH with recurrent or refractory pulmonary edema are considered as indications. Cardiac catheterization is a specialized investigation, not available in most of the hospitals managing these patients, hence requires transfer of a sick patient to a center performing CC. One key distinction from adults is the routine use of general anesthesia in infants and young children for catheterization. Respiratory acidosis from hypoventilation can worsen PVR and anesthetic gases can lower pulmonary arterial pressures affecting the validity of the results [78,79]. Complications such as death, vascular injury, arrhythmias and bleeding are around 7.3%, which is of concern [80]. Infants <6 months of age are at higher risk of complications including death (odds ratio 4.4 (1.88–10.35)) [80]. In young children undergoing CC, the incidence of death or need of extracorporeal membrane oxygenation (ECMO) was 3.5% [81], and the risk in premature infants was still higher (OR–4.95 (1.3–18.86)) [81]. Cardiac catheterization is an essential part of diagnosis and management of PH and performed in experienced centers for better outcomes.

## 4. Management of Infants with PH

### 4.1. General Measures

**Figure 4:**
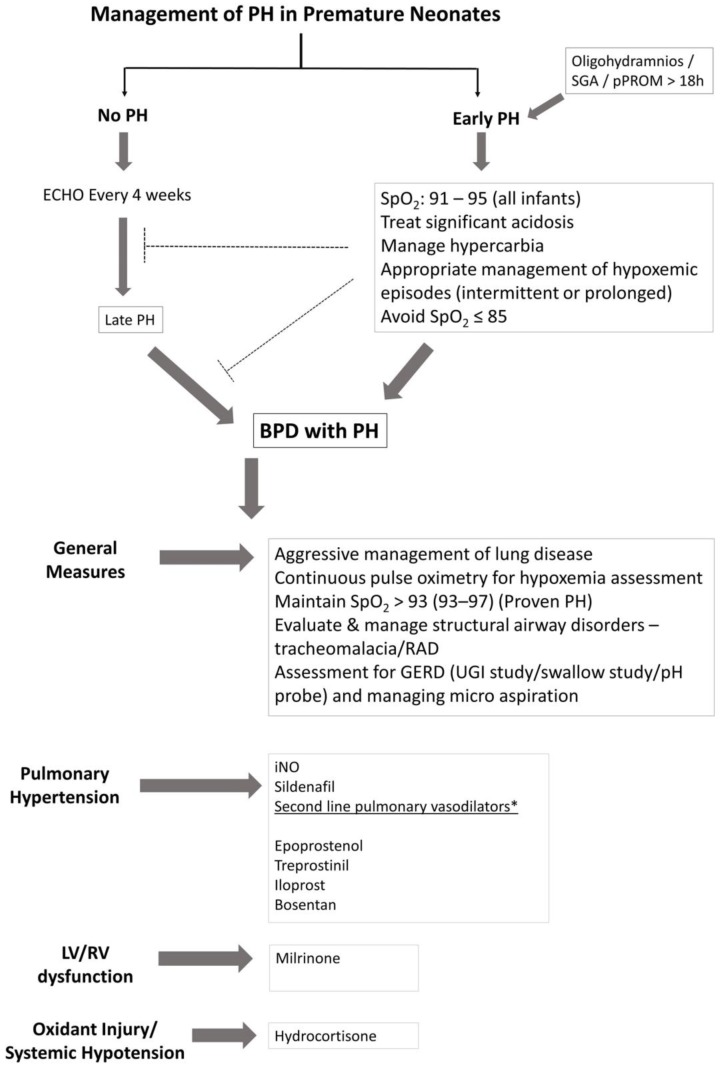
Management of pulmonary hypertension in premature neonates. Dotted lines indicate negative feedback loop on development of PH. RAD: reactive airway disease; UGI: upper gastro-intestinal series; OI: oxygenation index; * second line drugs are not well studied in neonates and infants with PH.

Treatment of pulmonary hypertension in infants and neonates has evolved in the past decade; nonetheless, the management decisions are often extrapolated from adult and pediatric studies. The treatment goals are directed towards improving hemodynamic profile (lowering PAP), delaying the progression of the disease, and preventing mortality in these infants. Severity of lung disease and infant’s nutritional status are closely linked to the progression of PH. Physiologic variations in SpO_2_ from intermittent hypoxemic episodes and/or hypercarbia are additional precipitating factors (Figure 4). Management of these factors may not only decrease BPD, but also help in slowing down the pathological process of pulmonary hypertension in these infants. However, this could be uniquely influenced by prenatal, genetic and epigenetic factors that may ultimately determine the progression of PH in these patients.

**Figure 5:**
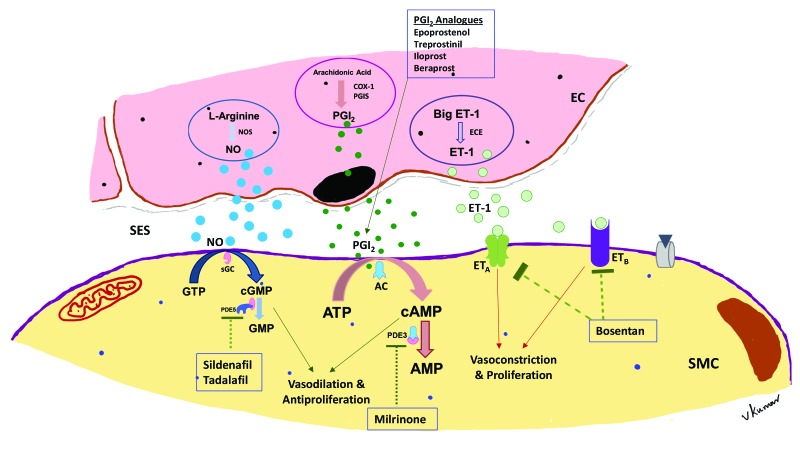
Schematic diagram of vascular mediators of the lung, their mechanism of action, interaction between the endothelial cell and the smooth muscle cell in the pulmonary vasculature, and its relationship to drugs used in the management of PH in BPD. The three predominant signaling pathways illustrated include: (i) nitric oxide—cGMP system; (ii) PGI_2_-cAMP system; and (iii) endothelin-1 system. NO: nitric oxide; PGI_2_: prostacyclin; NOS: nitric oxide synthase; EC: endothelial cell; ET-1: endothelin-1; ECE: endothelin converting enzyme; SES: subendothelial space; SMC: smooth muscle cell; PDE5: phosphodiesterase type 5; PDE3: phosphodiesterase type 3; ET_A_: endothelin receptor type A; ET_B_: endothelin receptor type B; AC: adenylate cyclase; sGC: soluble guanylyl cyclase; cGMP: cyclin GMP; COX1: cyclo-oxygenase type 1; PGIS: Prostaglandin synthase

Maintaining appropriate SpO_2_ and PaCO_2_ by relevant ventilation strategies would go a long way in amelioration of pulmonary hypertension in these infants. Maintaining SpO_2_ within a target range of 91–95% is crucial; however, it is practically difficult to achieve SpO_2_ in the desired range in a given patient at all times. The desired saturation target range is achieved, in less than 50% of the time despite overcoming the barriers for saturation targeting in premature infants [82]. Avoiding significant fluctuations in SpO_2_, not only helps in minimizing intermittent hypoxemic episodes, but also decreases the hypoxia–hyperoxia reoxygenation cycles of tissue injury. Fluctuating cycles of inspired oxygen may alter angiogenic gene expression [44]. The European Pediatric Pulmonary Vascular Disease Network recommends SpO_2_ of ≥93% in suspected PH and SpO_2_ of ≥95% in proven PH [83], however preductal SpO_2_ of >97% should be avoided. Persistent hypoxemic episodes (SpO_2_ ≤ 85%), should also be avoided particularly in growth-restricted infants and in infants with established BPD, as it may worsen vessel reactivity, promote vascular remodeling and exaggerate pulmonary hypertension. Managing hypercarbia (PaCO_2_ > 60 mmHg) is difficult in infants with BPD, and this may affect hypoxic pulmonary vasoconstriction, ventilation-perfusion mismatch and vessel reactivity. Nutritional status of the infant to meet adequate calorie intake for both energy expenditure and ongoing growth requires monitoring with guidance from a nutritionist. Noninvasive modalities of investigation such as CT and/or MRI are performed before subjecting the patient to a catheterization procedure. Appropriate management of associated cardiovascular anomalies along with pulmonary vasodilator therapy will help reduce mortality in these infants. As pulmonary vasodilators act via distinct signaling pathways (Figure 5), using them selectively or in combination may be of benefit in the management of PH in infants and children. Table 1 summarizes the commonly used vasodilators in the management of PH in infants.

### 4.2. Inhaled Nitric Oxide

Inhaled nitric oxide has been studied extensively and is the standard of care in the management of hypoxic respiratory failure (HRF) and persistent pulmonary hypertension of the newborn in term and late-preterm infants [84,85]. In animal models of lung injury, iNO promotes angiogenesis, decreases apoptosis, and reduces lung inflammation and oxidant injury [86,87,88]. As reduced number of alveoli, abnormal pulmonary vasculature, and inflammation characterize BPD, iNO was considered as the magic bullet to reduce BPD in premature infants. Several randomized control trials have evaluated the role of iNO in premature infants ≤34 weeks GA with varying results. Among the several randomized controlled trials conducted to date, neither rescue [89,90] nor routine use [91,92] of iNO improves survival in preterm infants with respiratory failure. The majority of evidence does not support treating preterm infants who have respiratory failure with iNO for the purpose of preventing or ameliorating BPD, severe intraventricular hemorrhage (IVH) or other neonatal morbidities [93]. In fact, early rescue treatment with inhaled nitric oxide may increase the risk of mortality and IVH in ELBW infants’ ≤1000 g [90]. Rescue trials with iNO were conducted prior to oxygenation saturation trials, which have demonstrated higher mortality in lower SpO_2_ group (85%–89%), especially in SGA infants [43]. This is the same group (SGA infants) also at increased risk for PH, and hence at increased risk for mortality. Despite direct beneficial effects on the brain in animal models [94,95], iNO has no demonstrable beneficial effect on neuro-developmental outcomes in premature infants [96,97].

Despite the absence of evidence of beneficial effects of iNO on preventing or decreasing BPD, iNO is extensively used in the management of PH associated with hypoxic respiratory failure (HRF), particularly when there is no response to other forms of therapy. Premature infants fall under either one of the two indications for initiation of iNO: moderate HRF with echocardiographic evidence of PH prior to initiation of iNO or severe HRF wherein iNO was initiated, as a desperate measure without a prior echocardiogram. Premature infants treated with iNO had a higher incidence of prolonged preterm rupture of membranes (pPROM) and oligohydramnios [21]. Preterm infants with HRF exposed to antenatal steroids and pPROM had improved oxygenation response to iNO and survival [21]. Antenatal betamethasone reduces oxidative stress and improves response of pulmonary arteries to vasodilators in lambs with PPHN [98].

Similar results were demonstrated in another study, wherein premature infants <32 weeks GA with either premature rupture of membranes (PROM) or prolonged PROM (pPROM), responded favorably to iNO following HRF with or without pulmonary hypertension [99]. Oxygenation index and ventilation requirements decreased shortly after initiation of iNO therapy in these infants. In addition, of all of infants with PPROM and PPHN, in the majority of them PPROM began before 24 weeks gestation and persisted for more than seven days. These findings suggest that in preterm infants with PPROM, oligohydramnios and pulmonary hypoplasia are additional contributing factors for the development of PH [22,99,100]. Targeting iNO use in this subset of preterm infants with severe respiratory failure after PPROM, may successfully treat the underlying respiratory pathology and improve further the ability to minimize disease and mortality in the preterm population [100]. Undetectable nitrite/nitrate levels in airway samples in infants with PPROM and PH suggest a deficiency of endogenous NO production and/or signaling defect playing a role in the pathogenesis of HRF with PH [99]. However, the role of NO signaling pathways is complex in the preterm lung and needs further study.

### 4.3. Sildenafil

Disruption of the NO-cGMP mechanism from factors related to preterm birth such as preterm rupture of membranes, growth restriction and lung injury have resulted in therapies targeting the NO-cGMP pathway [101,102]. Sildenafil, increases cGMP in the vasculature by inhibiting the enzyme phosphodiesterase 5 (PDE5) resulting in vasodilation and is widely used in the treatment of PAH in adults and children. Hyperoxia, commonly experienced by premature infants, may lower cGMP levels by increasing the expression and activity of PDE5 exacerbating PH [103]. The ease of administration and the beneficial effects particularly in infants on feeds makes oral sildenafil the preferred pulmonary vasodilator in infants with BPD and PH. Sildenafil is generally started at 0.5 mg/kg orally every 8 hours (q8) and titrated up to 2 mg/kg administered 6 hourly (q6) over 2 weeks to a daily maximum dose of 8 mg/kg/day. A significant reduction in estimated right ventricular peak systolic pressure is observed after initiation of sildenafil citrate, with the majority of infants showing no improvement in gas exchange at 48 h of treatment [104]. Most of these infants are on iNO prior to sildenafil treatment. In this particular study, sildenafil citrate was started at an oral dose of 0.25 to 0.5 mg/kg/day, given 6 hourly and titrated to systemic effects on systemic blood pressure or clinical reduction in respiratory support to a maximum dose of 6mg/kg/day (median age at initiation was 167 (83–307) days) [104]. Sildenafil treatment in patients with PAH secondary to BPD was associated with an echocardiographic improvement in the majority of patients, whereas clinical improvement was observed in a minority of patients [105]. Chronic sildenafil therapy is well tolerated, safe and effective in infants with chronic lung disease (CLD) and PH. In a retrospective study of 25 patients with CLD with PH, sildenafil (max dose of 8 mg/kg/day) initiated at a median of 171 days (median duration was 241 days), achieved hemodynamic improvement after a median treatment duration of 40 days, defined as a 20% decrease in pulmonary to systemic arterial pressure or an improvement in ventricular septal flattening [106]. Five patients died during sildenafil treatment, although none specifically from refractory PH [106]. Beneficial effects of chronic sildenafil therapy may include improvements in alveolarization and lung angiogenesis as demonstrated in rodent models [107].

Although sildenafil is well-tolerated, long-term outcomes from chronic therapy are unclear. A multi-center retrospective study demonstrated that sildenafil therapy does not increase the risk of retinopathy of prematurity in very low birth weight infants [108]. In 2012, the federal drug administration (FDA) released a strong warning against the chronic use of sildenafil for children with PAH (1–17 years), as an unexplained increase in deaths was noted in children on a higher dose of sildenafil compared to a low dose of sildenafil [109]. Consensus statement strongly recommended to adjust the dosage to 10 mg t.i.d. (8–20 kg body weight) or 20 mg t.i.d. (>20 kg BW) [110]. The FDA review was not applicable for short-term use of sildenafil in a critical care setting such as neonatal intensive care unit (NICU). It is common practice to treat PH in premature infants with sildenafil in NICUs and some of them are discharged home on chronic sildenafil therapy. Despite off-label use, the safety and efficacy of sildenafil in preterm infants with PH is not established. Long-term outcomes, especially relating to mortality in infants on sildenafil therapy is unknown. Hence, the use of sildenafil in premature infants with PH has to proceed with guarded optimism, pending future studies.

### 4.4. Prostacyclin Analogues

Prostacyclin (PGI_2_) increases cAMP by stimulating the enzyme adenylate cyclase within the vascular smooth muscle resulting in vasodilatation. Prostacyclin is reduced in children with PAH, and administering PGI_2_ or its analogues such as epoprostenol, iloprost, beraprost or treprostinil has been the mainstay in managing these patients. There is paucity of literature for many of these second line drugs, especially in premature infants with PH. The dose and the route of administration (Table 1, Figure 5) are specific to each prostanoid; however, these drugs have not been extensively studied, especially in infants and small children. Aerosolized prostanoids selectively dilates the pulmonary circulation and redistributes pulmonary blood flow away from non-ventilated regions of the lung [111]. Prostacyclin is well tolerated in recommended doses of 2 to 50 ng/kg/min; however, adverse events such as hypotension, cyanosis and feeding intolerance may occur [112]. Continuous intravenous epoprostenol (half-life: 2–3 min) is complicated by the need of central access, its associated catheter-related complications and the need for a specialized setting for its administration. Subcutaneous treprostinil is a long acting prostanoid (half-life: 4 h) with hemodynamic effects similar to epoprostenol. In five preterm infants with PH, treprostinil was not only safe, efficacious and well-tolerated but also resulted in improvements in right ventricular function, pulmonary pressures and decreasing respiratory support [113]. Inhaled treprostinil has been shown to improve exercise capacity with an acceptable safety profile in children with PAH [114]; however, its use in infants is still limited. Noninvasive ventilation and nebulized iloprost improved oxygenation and hemodynamics in ex-preterm babies with impending respiratory failure and PH [115]. Although prostanoids are used as primary agents for chronic PH management in adults and children, they are add-on therapies to the primary agents that are either iNO or sildenafil in infants with PH. Studies need to address both the efficacy and safety of these agents in infants.

### 4.5. Endothelin Antagonists

Endothelin-1 (ET-1) mediates vasoconstriction and smooth muscle cell proliferation contributing to the development of PAH. The actions of ET-1 are mediated mainly through the ET_A_ receptor. Bosentan, a dual endothelin receptor antagonist, is an oral antagonist of both ET_A_ and ET_B_ receptors. It is the most widely studied endothelin receptor antagonist (ERA) in children. However, studies in infants are mostly limited to term infants. In a randomized trial, bosentan was superior to placebo in term infants with PPHN [116]. Assessment of favorable outcome was by improving oxygenation index, decreasing PAP on echocardiogram and absence of side effects. There were no drug-related clinical or laboratory adverse events in these infants. In a recent randomized trial, bosentan although well tolerated did not improve oxygenation or other outcomes [117]. Delayed absorption or severe illness may have contributed for the lack of response to bosentan in these infants. Administration of bosentan is mostly in combination with other agents in premature infants with PH. Potentially serious hepatotoxicity and teratogenicity with bosentan requires close monitoring and cautious use.

### 4.6. Hydrocortisone

Hydrocortisone decreases hyperoxia-induced PDE5 activity and markers of oxidative stress in lambs with PPHN ventilated with 100% O_2_ [118]. Actions of hydrocortisone are mediated by attenuating the reactive oxygen species induced NF-κB activity, thereby decreasing PDE5 activity [119]. Hydrocortisone not only acts by its anti-inflammatory action, but also by decreasing the right to left shunting via the ductus by increasing systemic blood pressures. However, clinical studies have not been convincing, and conflicting data have been reported in the literature on the efficacy of steroids in meconium aspiration syndrome in term infants [120,121]. Despite the anecdotal use of hydrocortisone as a last resort in both term and premature infants with PPHN, there is paucity of evidence to support the use of steroids in the management of pulmonary hypertension, especially in premature neonates.

### 4.7. Milrinone

Milrinone is a phosphodiesterase III inhibitor, which increases the bioavailability of cAMP and is shown to improve pulmonary hemodynamics in animal experimental models [122]. Milrinone is widely used in congenital diaphragmatic hernia (CDH) infants in the management of PH with cardiac dysfunction [123]. Milrinone may be a useful adjuvant therapy for premature infants with echocardiography findings of PH and/or RV dysfunction [124]. Premature infants who respond sub optimally to iNO may have left ventricular dysfunction; milrinone by increasing contractility and decreasing afterload improves cardiac function and decreases pulmonary vascular resistance. Systemic hypotension is a serious concern with intravenous milrinone and hence it has to be used with caution.

### 4.8. Combination Therapy

The goal of combination therapy is both to maximize efficacy and to minimize toxicity. Agents that inhibit degradation of cGMP (PDE-5 inhibitors) may act synergistically with iNO. Similarly inhibiting PDE-3 an enzyme that degrades cAMP might augment the response to PGI_2_. Intravenous milrinone (↑ cAMP) has been used in combination with iNO (↑ cGMP) in the management of PH in neonates [125]. Orally administered sildenafil and inhaled iloprost combination was successful in management of PH in a preterm neonate with BPD and RSV infection [126]. Combination therapies are required in patients on prolonged vasodilator therapy or weaning from iNO treatment and in patients not responding to standard measures.

### 4.9. Future Directions

**Figure 6:**
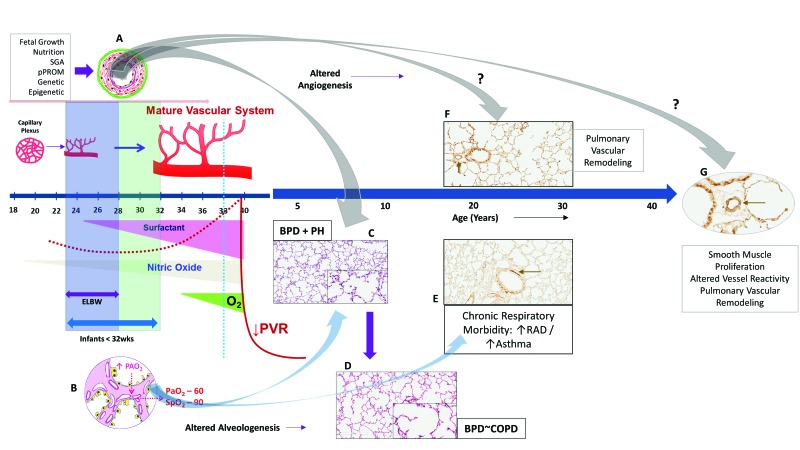
Fetal programing, preterm birth and pulmonary hypertension. Extremely Low Birth Weight (ELBW) infants born between 23 and 28 weeks (grey zone) are at risk for BPD and PH, as determined by lung development, fetal growth, and epigenetics. The degree of altered angiogenesis and alveologenesis determine the severity of BPD and/or PH. (**A**) Cross-section of pulmonary arteriole at birth in premature infant; (**B**) alveoli exposed to high PO_2_, hence higher arterial PO_2_; (**C**) BPD in mice exposed to O_2_ from P3–P14; (**D**) lungs similar to COPD/emphysema in adult mice; (**E**) airways with smooth muscle hypertrophy on SMA staining (arrow) in 3-month adult mice. Premature infants with BPD are at increased risk for RAD, bronchial hyper-responsiveness and asthma; (**F**/**G**) pulmonary arterioles with thickened walls on SMA staining suggesting smooth muscle proliferation (adult mice aged 3 to 9 months). The risks of preterm birth associated with late onset pulmonary hypertension in children and adults is not known in humans.

Various prenatal and postnatal factors regulate the extent of alveolar and vascular dysplasia to determine the severity of pulmonary hypertension on the background of the immature lung in the preterm neonate. We are beginning to understand the role of fetal origins of pulmonary vascular disease, aggravated by postnatal factors such as infection, ventilation and oxygen. The implications of intrauterine growth restriction on vessel reactivity and late onset pulmonary hypertension in adults in still not clear (Figure 6). We observe some of the most severe forms of PH in the NICU, infancy and in early childhood. However, the effects of altered lung development may be more subtle and could have consequences in adults. Preterm infants have altered airway function as adults [127] and may have pulmonary vessel remodeling that is different from infants born at term. The impact of epigenetics on the pulmonary vasculature, screening methodologies in the diagnosis of PH and studies specifically addressing management of PH in infants has to be explored further.

## Figures and Tables

**Table 1:** Vasodilators in the management of pulmonary hypertension in infants.

Drug	Dosage/Route of Administration	Common Adverse Effects
Nitric Oxide	Inhaled 5–20 ppm (OI > 20); Wean iNO—FiO_2_ < 60%; PaO_2_ > 60 mmHg; Keep SpO_2_ ≥ 91; Infants on chronic iNO therapy—wean last 5 ppm gradually to ↓ rebound PH	Monitor methemoglobin during use
PDE5 Inhibitor—Sildenafil	Oral—0.5 mg/kg q8–6 ↑ to 2 mg/kg q8–6 over 2 weeks IV (continuous infusion): 0.4 mg/kg over 3 h (LD); Infusion—0.07 mg/kg/h	Systemic hypotension; watch for worsening oxygenation due to vasodilation of unventilated areas of the lung; flushing, diarrhea, nasal congestion, priapism
Prostanoids *—Epoprostenol	IV/continuous Aerosolization—2 ng/kg/min ↑ to 20–50 ng/kg/min	Systemic hypotension, nausea, vomiting, flushing, diarrhea, thrombocytopenia, bloodstream infection
Treprostinil	Subcutaneous—1.5 ng/kg/min ↑ to 20–40 ng/kg/min; Inhaled—3–9 breaths (6 µg/breath) q6	Infusion site pain, site infection, flushing, diarrhea, nausea, jaw pain, bloodstream infection
Iloprost	Inhalation: 1–2.5 µg/kg q2–4 h	Cough, syncope, hypotension, flushing, headache, trismus
PDE3 Inhibitor—Milrinone	IV—50 µg/kg (LD) over 1–2 h; Infusion—20–75 µg/kg/min	Hypotension, tachycardia, arrhythmias, thrombocytopenia, low potassium, bronchospasm
Endothelial Receptor Antagonist—Bosentan	Oral: 1 mg/kg q12	Hypotension, flushing, hepatotoxicity, anemia, thrombocytopenia, teratogenesis

IV: intravenous; SC: subcutaneous; LD: loading dose. Sildenafil is commonly administered per oral (PO) occasionally IV. * Second line drugs are not well studied in neonates and infants with PH.
